# Influence of the pulse repetition rate on the acoustic output of ballistic pressure wave devices

**DOI:** 10.1038/s41598-022-21595-5

**Published:** 2022-10-27

**Authors:** Nina Reinhardt, Jens Wegenaer, Matías de la Fuente

**Affiliations:** Chair of Medical Engineering, Helmholtz-Institute for Biomedical Engineering, RWTH Aachen University, Pauwelsstr. 20, 52074 Aachen, Germany

**Keywords:** Biomedical engineering, Medical research, Preclinical research

## Abstract

Ballistic devices that generate radial pressure waves are used for the treatment of different therapeutic indications. In order to assess the effectiveness of these devices and to interpret and transfer the results of clinical trials, it is important to know their acoustic output. In this paper, two ballistic devices and their reproducibility at different clinically relevant settings were investigated in the same in-vitro test setup. Pressure curves were measured in water at different intensity levels and pulse repetition rates. The sound field parameters (peak pressures, positive pulse intensity integral) were calculated from the pressure curves. Additionally, the surface velocity of the applicator was determined in air using a vibrometer. Both devices show a good pulse-to-pulse reproducibility. While the peak maximum pressure and the positive pulse intensity integral decrease only slightly (p_max_ up to 12%, PII^+^ up to 18.8%) comparing 1 Hz and 25 Hz for one device, they drop sharply (p_max_ up to 68.4%, PII^+^ up to 90.2%) for the other device comparing 1 Hz to 21 Hz. The same effect was observed in the vibrometer measurements. The results show that with increasing pulse repetition rate the stability of the parameters varies between different devices. Hence, all sound field parameters should be compared before transferring settings from one device to another.

## Introduction

Radial pressure waves generated by ballistic devices are used for non-invasive treatment in various orthopaedic and musculoskeletal indications. A projectile is accelerated onto an applicator at high speed by means of compressed air or an electromagnetic field. The produced pressure wave is transmitted to the target area of the patient by using coupling gel and the energy density decreases proportional to the square of the penetration depth. These devices, also known as radial shock wave therapy (rESWT) devices, can be characterized by acoustic output measurements as described in the IEC 63045:2020 standard.

The correlation between the shock wave parameters and the therapeutic effect is not yet fully understood. To transfer the results of clinical studies conducted with one device to another and to evaluate their effectiveness, the physician needs to know the parameters used during the treatment. In the treatment guidelines^1^, different input pressures are recommended for different indications, but the resulting sound field depends on the applicator and the device used. Therefore, it is necessary to determine the acoustic output of the devices at clinically relevant settings. The existing devices can treat at pulse repetition rates (PRR) of up to 25 Hz.

Two methods are described in the IEC 63045:2020 standard: the wet test bench and the dry test bench. Pressure measurements of ballistic devices have been performed in water (wet benches)^2–5^ and behind a silicon pad (dry benches)^6–8^. Wet test benches have the advantage that the spatial distribution of the sound field can be determined. However, it is challenging to evaluate devices at high PRRs as the water tends to cavitate and disturb measurements. In contrast, dry test benches can be used without cavitation, but are limited to few measurement spots. Furthermore, the acoustic output of ballistic devices can be determined by means of a laser vibrometer. This method was used to determine the velocity of the applicator surface^9^. Although conclusions about the generated pressure field can be drawn from these measurements, the measured values cannot be directly transferred to sound field parameters.

In addition to the sound field parameters, their reproducibility is also important. This can be assessed on the one hand by the pulse-to-pulse variation and on the other hand by the continuity of the generated pressure pulses over the pulse repetition rate, i.e. the frequency at which the pulses are fired. Cleveland et al. recorded stable peak pressures for all PRRs in water evaluating one device^3^. Ueberle and Jamshidi Rad showed in a dry test bench that the measured pressure can vary significantly with increasing PRR depending on the device^6^. In force measurements with a piezoelectric load cell, a decrease in output with increasing PRR was observed^7^.

In clinical use, besides the number of sessions and the number of pulses, especially the driving pressure of the projectile and the PRR can be adjusted individually. The aim of this work was to investigate the reproducibility of the acoustic output generated by two ballistic devices in a water test bench at different PRRs and different driving pressures of the projectile. To exclude an influence of the setup, follow up measurement using a laser vibrometer were performed.

## Materials and methods

**Figure 1:**
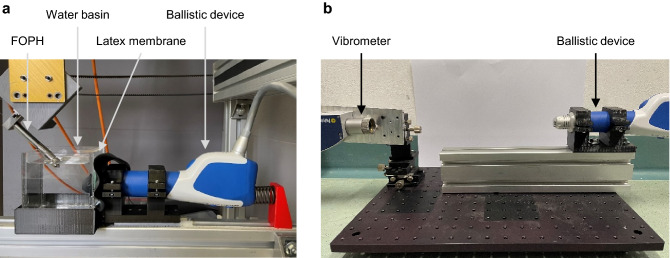
Test setups. (**a**) Pressure measurements. (**b**) Vibrometer measurements.

Two ballistic devices generating radial pressure waves were evaluated in this study: DolorClast Radial Shock Waves with Blue handpiece and 15 mm applicator (EMS Electro Medical Systems S.A., Nyon, Switzerland) (DUT1) and MasterPuls 200 Ultra with Falcon handpiece and 15 mm applicator (Storz Medical AG, Trägerwilen, Switzerland) (DUT2). The influence of the PRR on the pressure curves and the surface velocity of the applicators was determined. The pressure curves were measured in a hybrid test bench according to the IEC 63045:2020 standard. The test setup was described previously^10^. Measurements were conducted using a fibre optic pressure hydrophone (FOPH 2000, RP acoustics e.K., Leutenbach, Germany), which was positioned in an angle of 45° to the beam axis. The ballistic devices were coupled to a small water bath using a 1 mm latex membrane and ultrasound gel (Fig. 1a). The ultrapure degassed water had a temperature of 23.1 ± 0.7 °C. The contact pressure was applied using a preloaded spring. For each device, different pressure levels driving the projectile were investigated: 1, 2, 3, 4 bar. For DUT2, the maximum pressure level of 5 bar was examined additionally. The pressure curves were measured at PRRs of 1, 5, 10, 15 and 20 Hz as well as the maximum PRR of each device (DUT1: 25 Hz, DUT2: 21 Hz).

The hydrophone output was measured using a DPO 2024 oscilloscope triggered on the falling signal amplitude. At each setting, every 10th shot was measured and 10 pressure curves were recorded on the beam axis at 2 mm distance to the latex membrane. Before calculating the parameters of the pressure curves, each curve was filtered with a 1 MHz Butterworth low pass filter. The peak maximum pressure (p_max_), the first peak negative pressure (p_min_) and the positive pulse intensity integral (PII^+^), also known as positive energy density, were calculated according to the IEC 63045:2020 standard. For the calculation of PII^+^ the first positive pulse was considered. The pulse-to-pulse reproducibility was examined comparing all curves measured at the same setting. For better visualization, the filtered curves were averaged.

Measuring shock waves in a water bath at high pulse repetition rates can result in a high amount of cavitation, which may falsify the pressure measurement. In order to exclude this, measurements were repeated using a vibrometer. A vibrometer (Polytec CLV 2534, Polytec GmbH, Waldbronn, Germany) was used to determine the surface velocity of both applicators in air. The laser beam of the vibrometer pointed to the centre of the applicators (Fig. 1b). Each device was evaluated at its maximum pressure level (DUT1: 4 bar, DUT2: 5 bar) at different PRRs (1, 5, 10, 15 and 20 Hz). The velocity of the applicators was recorded three times at each setting. Each curve was high pass filtered with 10 kHz cut-off frequency and the peak maximum velocity was calculated.

## Results

**Figure 2:**
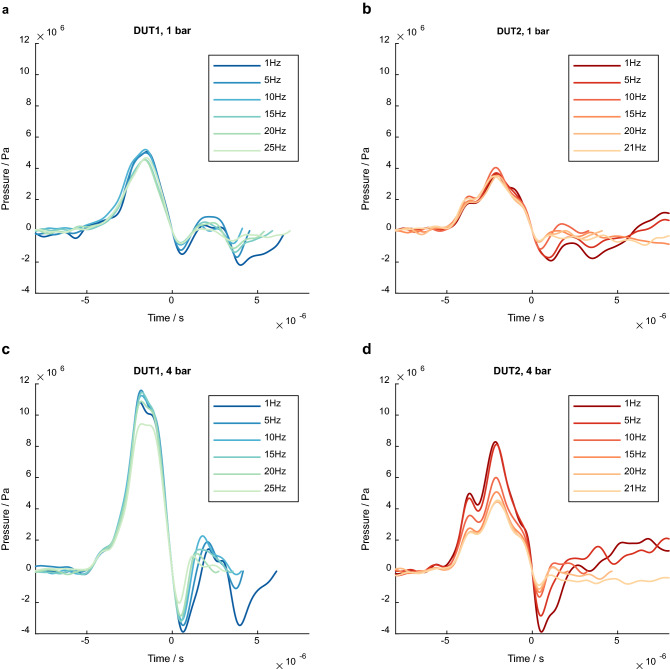
Examples of the filtered mean pressure curves at different pulse repetition rates at 1 bar (**a**,**b**) and at 4 bar (**c**,**d**).

The first peaks of the filtered and averaged pressure curves show a good stability for all PRR at pressure level 1 bar for both devices (Fig. 2a,b). At a high pressure level of 4 bar, the first peak of DUT1 drops slightly at the highest PRR, at all other PRRs the waveform of the first positive peak remains constant (Fig. 2c). For DUT2, a decrease in amplitude with increasing frequency was observed (Fig. 2d). However, the general waveform remains similar for both devices in all settings. A decrease in the first negative pressure peak with increasing PRR was observed at all pressure levels examined.

**Figure 3:**
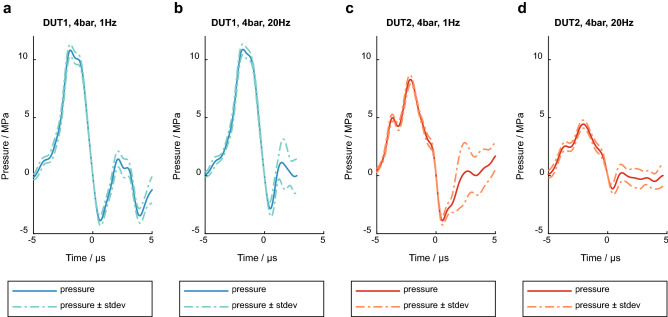
Examples of mean pressure curves and standard deviation of both devices at 4 bar and different PRR. (**a**) DUT1, 1 Hz. (**b**) DUT1, 20 Hz. (**c**) DUT2, 1 Hz. (**d**) DUT2, 20 Hz.

**Figure 4:**
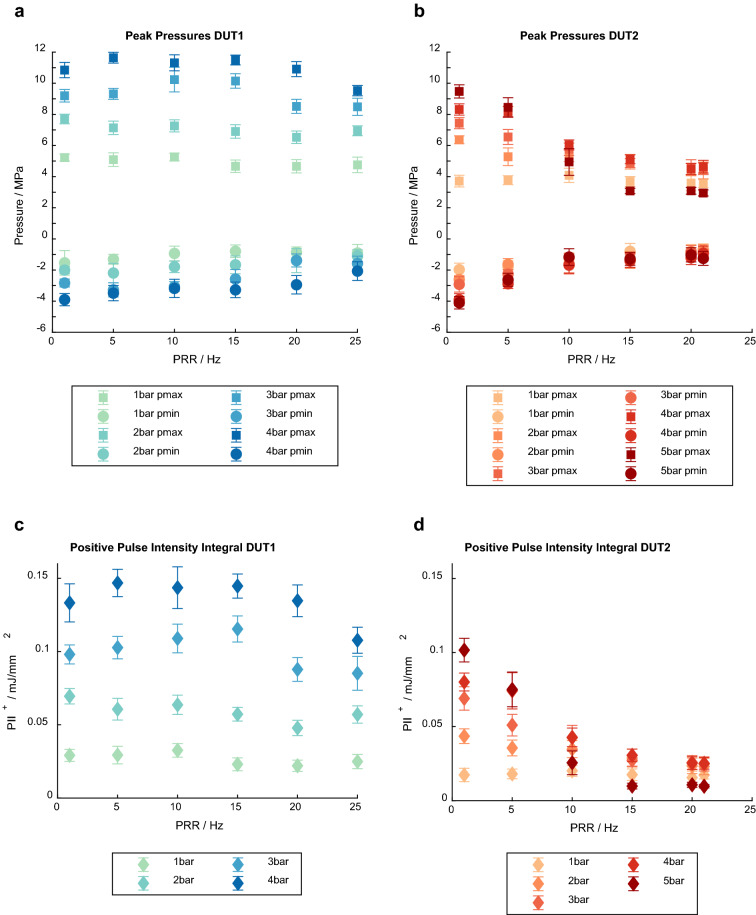
Pressure curve parameters. Measured peak maximum and minimum pressures (p_max_, p_min_) of both devices at different pulse repetition rates and intensity levels (**a**,**b**); Positive pulse intensity integral (PII^+^) of both devices at different pulse repetition rates and intensity levels (**c**,**d**).

All pressure curves of both devices show good pulse-to-pulse reproducibility for the first positive and first negative peak with a very small standard deviation between the results of the individual measurements (Fig. 3). This is also underlined by the values determined for the characteristic parameters, p_max_, p_min_ and PII^+^ (Fig. 4).

The mean peak maximum pressure of DUT1 decreased by 0.4 MPa (1 bar)–1.3 MPa (4 bar) from 1 to 25 Hz (Fig. 4a), corresponding to a loss of 7.7–12%. The highest mean p_max_ generated by DUT1, 11.6 MPa, was reached with 4 bar driving pressure and a PRR of 5 Hz. The mean p_max_ of DUT2 decreased by 0.1 MPa (1 bar)–6.5 MPa (5 bar) with increasing PRR from 1 to 21 Hz (Fig. 4b), which is equivalent to a loss between 2.7 and 68.4%. The mean of the first peak tensile pressure decreased with increasing PRR for both devices at all pressure levels. In DUT1, the smallest decrease was observed at 1 bar (0.6 MPa) and the largest decrease at 3 bar (1.8 MPa), while in DUT2, the smallest decrease was found at 1 bar (1.3 MPa) and largest decrease at 4 bar (3.0 MPa).

The PII^+^ of DUT1 stayed in the same range at the lower pressure levels (1 bar, 2 bar) for all PRRs (Fig. 4c). It increased slightly at the higher pressure levels from 0.098 mJ/mm^2^ (3 bar) and 0.133 mJ/mm^2^ (4 bar) at 1 Hz to 0.115 mJ/mm^2^ (3 bar) and 0.145 mJ/mm^2^ (4 bar) at 15 Hz and then decreased slightly to 0.085 mJ/mm^2^ (3 bar) and 0.108 mJ/mm^2^ (4 bar) at 25 Hz. These results correspond to a loss in PII^+^ of 13.3% and 18.8% between the lowest and the highest PRR at 3 bar and 4 bar, respectively. At pressure level 1 bar, the positive pulse intensity integral of DUT2 stayed constant at PRRs 1 Hz and 21 Hz (0.017 mJ/mm^2^) and was in the same range for the other PRRs (Fig. 4d). At all other pressure levels the PII^+^ decreased with increasing PRR. The most significant drop in PII^+^ was observed at pressure level 5: It decreased from 0.102 mJ/mm^2^ at 1 Hz PRR to 0.010 mJ/mm^2^ at 21 Hz PRR, which corresponds to a loss of 90.2%.

**Figure 5:**
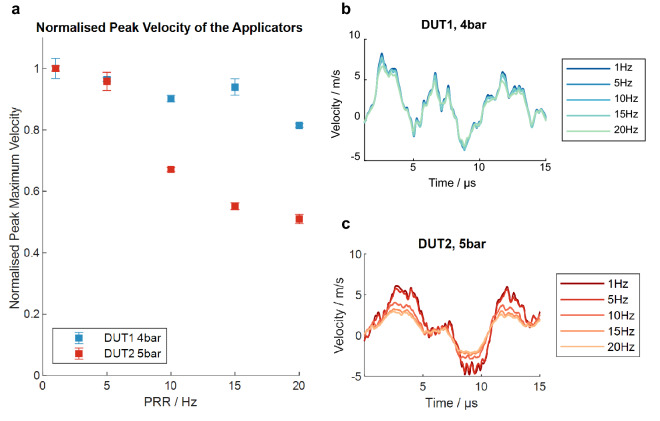
Vibrometer measurements. (**a**) Normalised peak maximum velocity measured for DUT1 and DUT2 at different pulse repetition rates and maximum pressure level. Mean velocity curves measured for DUT1 (**b**) and DUT2 (**c**) at different pulse repetition rates and maximum pressure level.

The vibrometer measurements at the highest pressure level of each device showed a dependency of the applicators’ surface velocity on the PRR (Fig. 5a). The mean peak maximum velocity of the applicator of DUT1 decreased by 18.5% at a PRR of 20 Hz compared to 1 Hz. For DUT2, a 49.1% drop in applicator velocity was observed when increasing the PRR from 1 to 21 Hz. The general waveform of the velocity in air matches the pressure curves in water well in terms of frequency (Fig. 5b,c). However, the speed curves oscillate longer with constant amplitude.

## Discussion

The measurements described in this work showed differences in the stability of two ballistic pressure wave devices at high PRRs. The effect was particularly clear in high driving pressures of the projectile and was observed in both, the pressure field and the applicator velocity.

Both devices showed a good pulse-to-pulse reproducibility for all settings investigated. The pulse repetition rate had a higher impact on the pressure curves generated by DUT2, while pressure curves generated by DUT1 were more stable. A difference between two devices in terms of the constancy of pressure curve parameters with increasing pulse repetition rate was also observed in a dry bench^6,11^. We were able to confirm the results from the dry bench by measurements in water and additionally showed the dependence on the input pressure. In the hybrid test bench described in this work, p_max_ and PII^+^ generated by DUT2 decreased by up to 68.4% and by up to 90.2%, respectively, with the PRR increasing from 1 to 21 Hz. The pressure curves of DUT1 showed less variability with losses in p_max_ of up to 12% and losses in PII^+^ of up to 18.8% comparing 1 Hz and 25 Hz PRR. Other studies with devices comparable to DUT1 have also shown good stability even at high PRRs^3,10^. Cosoli et al. found a PRR-dependent decrease of the output of another ballistic device in force measurements with a piezoelectric load cell^7^. They attribute it to a too slow refilling of the pressure reservoir. Differences in the air flow management systems of the two devices investigated in our study are a possible explanation for their different performance at high PRRs.

For both devices investigated in this study, the negative pressure was reduced with increasing PRR. One possible reason for this may be cavitation clouds, which can become larger and more persistent with increasing PRRs. This relationship between PRR and cavitation has been described in literature for focused shock waves^12–14^. Moreover, it has been shown for focused shock waves, that the negative pressure phase is damped by cavitation^12^.

The hybrid test bench used here can be used to determine the positive peak pressure as well as the first negative peak pressure and the positive pulse intensity integral at all clinically relevant pulse repetition rates. However, it is not suitable for calculating the total PII due to interferences in the further course of the signal. Furthermore, the optical fibre was positioned manually in this study. Therefore, the absolute values of the different devices are not directly comparable.

The vibrometer measurements are comparable to the hydrophone measurements in terms of the frequency of the curves. The longer lasting oscillation with higher amplitude can be explained by the lower damping in air compared to water. However, the results of the vibrometer measurements at the highest pressure levels (4 and 5 bar) underline the tendencies that were also observed in the pressure measurements. DUT2 shows a stronger decrease (49.1%) in applicator velocity than DUT1 (18.5%) comparing PRRs 1–20 Hz.

The output of the two devices investigated in this study and its stability are not comparable, especially at high pressures and high PRRs. From a clinical perspective, the results show that reliance on manufacturer-reported parameters at a PRR of 1 Hz can lead to a huge overestimation of treatment parameters at higher PRRs. The PII is the most commonly used parameter by clinicians to estimate the therapeutic effect. This study showed a considerable decrease in PII^+^ for DUT2 at high PRRs. Consequently, using this device at 5 bar and 21 Hz results in a comparable PII^+^ to using it at 1 bar and 1 Hz. The results highlight the importance of measurements done at clinically relevant repetition rates for ballistic pressure wave devices. Since the stability of the parameters varies between different devices, transferring treatment settings from one device to another is not recommended without comparing the parameters at all settings.

In future studies, additional devices should also be evaluated. Especially to better compare the results of clinical studies, a database with the input parameters and the resulting sound field parameters of as many devices as possible would be beneficial. We have shown a method that can be used to do this evaluation.

For a comprehensive evaluation, the entire sound field should be measured and a set of all sound field parameters should be determined in the future. Such a comprehensive parameter set could be used to correlate sound field parameters and their effects on tissue. Cavitation measurements can be performed for further assessment of the devices as well as the test bench.

## Data Availability

The datasets generated during and/or analysed in this study are available from the corresponding author upon reasonable requests.
